# Collective efficacy, self-efficacy, and socio-occupational wellbeing: a quasi-experimental study on an intervention based on the development of collective competences in a blended-learning context

**DOI:** 10.3389/fpsyg.2025.1462937

**Published:** 2025-11-14

**Authors:** María Lourdes Campos, Axel Bascur

**Affiliations:** Department of Psychology, Universidad de La Serena, La Serena, Chile

**Keywords:** blended-learning, collective efficacy, quasi-experimental, self-efficacy, socio-occupational wellbeing

## Abstract

**Background:**

Although innovation in the workplace seeks to improve the productivity and quality of life of members of organizations, the evidence of a low adoption of new strategies and technologies in companies highlights the need to find effective methods for the implementation of these strategies. In this context, the objective of our study is to verify the effect of an intervention with a blended- learning strategy, with a focus on the development of collective competencies, on the variables of self-efficacy, collective efficacy and socio-occupational wellbeing.

**Method:**

We employed a quasi-experimental pre-test/post-test design. The study variables were evaluated using a non-probability, purposive sample of 110 workers of a Chilean mining company. The intervention group was composed of voluntary participants, same as the control group, who only completed the evaluations. The intervention was conducted over a four-month period, and consisted in 5 in-person training workshops, in parallel with a complementary, continuously open online courses. The content of the training program focused on improving collective management, group synergy, collaborative problem-solving and communicative strategies.

**Results:**

The analysis shows that the collective competences intervention with the blended-learning program had a positive effect on the variables of self-efficacy, collective efficacy, and socio-occupational wellbeing and associated dimensions from the quasi-experimental group.

**Conclusion:**

The results of this study suggest that intervention based on the development of collective competences in a blended-learning context have a positive impact in the self-efficacy, collective efficacy and the social wellbeing on workers in the occupational context.

## 1 Introduction

Recent evidence indicates that innovation in the workplace is a developing field of research, where a growing body of findings and practical implementations is being constructed. These advances, which stem from the work done in various productive and disciplinary spheres, have a common objective which the literature unambiguously delineates: first, to improve organizational performance by increasing flexibility, productivity, or efficiency; second, to increase worker quality of life by developing competences, preventing stress, and giving meaning to one’s work life (Rus et al., 2020).

As these findings indicate, it is essential to build competences related to productivity and wellbeing in order to attain said goals.

Nevertheless, it has been observed that labor systems exhibit low rates of adoption of new work and organization methodologies. Studies on this issue show that, after insufficient funding, the main reasons for this limited implementation of innovations are worker and union resistance, administrative barriers, a lack of network-based coordination, perceived risks, a lack of incentives, the absence of qualified personnel, a short-term mentality, and a lack of an innovation-oriented culture (Gruenhagen and Parker, 2020). As the authors note, this problem highlights the absence of suitable innovation strategies which are able to adequately address the barriers present in organizations and workplaces.

It is with respect to this issue that, in Chile, despite the large sums devoted to boosting productivity, innovation, and human capital development in organizations, no significant improvements in productivity have been observed. As pointed out in the latest report issued by the National Evaluation and Productivity Commission (Comisión Nacional de Evaluación y Productividad, CNEP), the aggregated projection of national productivity rate fell from −1.8% to −2.4%, a deficit that, for this agency, is part of a decade-plus stagnation trend that illustrates the non-existent increase in aggregated productivity, stressing the need to generate remedial measures (Comisión Nacional de Evaluación y Productividad [CNEP], 2024).

In addition to the above, despite the reduction in unemployment rates in Chile (Comisión Nacional de Evaluación y Productividad [CNEP], 2024), several relevant socio-occupational problems remain. These include high absenteeism levels for medical reasons, with 7.8 million medical leave certificates being issued in 2023, of which 31.7% involved diagnoses of mental disorders (Superintendencia de Seguridad Social [SUCESO], 2024). Likewise, studies on psychosocial risk conducted using a sample of over four thousand workplaces and more than 280 workers indicate that nearly one third of these workplaces exhibit a medium level of psychosocial risk (31.6%), while 2.2% show a high level. In the latter, workers are liable to a range of physical and mental pathologies (Superintendencia de Seguridad Social [SUCESO], 2022).

In this context, marked by barriers to implementation and the need to introduce innovations aimed at increasing productivity and socio-occupational wellbeing, it is necessary to prioritize interventions with an approach that is contextualized, systemic, beneficiary-focused, and driven by companies’ operational needs without neglecting intangible factors like workplace climate, organizational culture, and administrative orientation (Gruenhagen and Parker, 2020). To meet these conditions, intervention methodologies should be implemented which seek to develop human and social capital and facilitate the construction of more solid workplace communities, and be designed to promote teamwork and collaboration, acknowledging the importance of workers as fundamental assets in efforts to improve productivity and wellbeing (Adams, 2020; Ehsan and Spini, 2020; Muzard, 2012; Valencia, 2005). In this regard, research shows that the interaction between individual and collective competences are necessary for the action and innovation system to succeed, since each individual activates and strengthens the competences of other actors (Fernández, 2006).

In parallel, researchers have drawn attention to Information and Communication Technologies (ICTs) as a path to improvement whose implementation has several advantages. The introduction of ICTs into organizations, apart from being linked to an overall increase in productivity (Hervas-Oliver et al., 2017), stands out due to their specific benefits, like the operational flexibility that they instill into organizations and their ability to rebuild work groups; the reduction in administrative needs when conducting certain actions or making certain decisions; and the increase in opportunities for employees to be autonomous and develop their skills within the context of their job and the organization (Medzo-M’engone et al., 2019). As noted by the Comisión Nacional de Evaluación y Productividad [CNEP] (2024), recent technological advances and the major expansion of remote work stress the importance of reviewing the available evidence and designing strategies (and policies) that make it possible to take advantage of these developments with a view to improving work efficiency. This agency asserts that interventions aimed at developing competences and innovations in the social and productive spheres of an organization will benefit from the use of technological and digital solutions, which can make it easier to address individual, communicational, and organizational needs present in occupational settings (Mishra and Koehler, 2006). This information suggests that training strategies that employ these technologies –e.g., e-learning, blended learning, or flipped classrooms– can indeed be successful (Garrison and Kanuka, 2004; Means et al., 2010).

Lastly, the global nature of today’s world demands permanent adaptation to changes in the occupational field, which emphasizes permanent learning and adaptation capabilities (Rakowska and de Juana-Espinosa, 2021). The rapid advancements in technology and the increasing interconnectedness of global markets necessitate that organizations remain agile and responsive to shifts in their operational landscapes. Organizations must invest in innovative training and development programs, such as mentoring, job rotation, and the use of advanced digital learning platforms, to ensure their workforce is prepared to meet current and future challenges (Cascio and Montealegre, 2016).

Based on this background information and the theoretical aspects articulated above, it can be stated that an intervention with a psychosocial focus, aimed at developing the collective competences of the members of an organization, will have positive effects on individual and collective capacities to attain productive goals and will also have a positive impact on the level of socio-occupational level of wellbeing perceived by organization members. Such an intervention must integrate digital implementation modes that facilitate the transference and benefits associated with the use of ICTs; therefore, we chose blended learning as the most suitable strategy. We defined three variables associated with the impact of an intervention targeting these characteristics: self-efficacy, as an indicator associated with the evaluation of individual performance within the context of organizational; collective efficacy, as an indicator linked to the development of collective competences in work settings; and socio-occupational wellbeing, as an indicator of workers’ perceived wellbeing when they evaluate the social conditions of their workplace, the quality of interpersonal interactions and relationships, and their understanding of social behaviors within the organization.

As the intervention proposes the development of collective competences, we first established the concept of “competence” as the capacity to apply an organized set of knowledge, skills, and attitudes within a specific context, making efficient use of the available resources (Mulder, 2017; Le Deist and Winterton, 2020). This constitutes a set of elements needed to perform functions and tasks successfully in the workplace, identifying performance criteria to evaluate their execution. Cheetham and Chivers (2001) highlight that competences marshal resources relevant to the context of operation, entailing a “contextualized know-how” that makes it possible to solve problems in complex work settings. These competences integrate conceptual, procedural, and attitudinal knowledge, involving reflection on the learning process.

We define collective efficacy as an indicator associated with an efficacy level different from self-efficacy, since it comprises a specific group of interpersonal and collaborative competences involved in the combined work of an organizational collective. According to Bandura (1997), collective efficacy refers to a group’s shared belief in its conjoint capabilities to organize and execute courses of action required to produce given levels of attainment. Collective competences entail constant collaboration and coordination within a group in order to achieve common goals (Stajkovic et al., 2009). These competences can be defined as the set of knowledge and skills shared by a group, used to address problems and attain shared objectives (Salas et al., 2008). In addition, the literature indicates that these competences are only utilized when the members of an organization coordinate their efforts to solve specific challenges, which stresses the importance of interpersonal skills as well as their collective nature (DeChurch and Mesmer-Magnus, 2010). The effectiveness of collective competencies rely heavily on the interplay of individual contributions within a group setting, highlighting the necessity for strong interpersonal relationships and collaborative dynamics (Rosen et al., 2020). Within the context of work and organizations, we employed a model that defines collective efficacy through four dimensions (Campos et al., 2020). The first, group competence/positive (GC+), can be defined as subjects’ positive assessment of the willingness, capacity, and conviction of the group to apply knowledge, skills, attitudes, or exchanges within the context of a participation that is active, collaborative, and focused on common outcomes. The second dimension, group competence/negative (GC−), can be defined as the negative assessment of these group characteristics, indicating that the subject perceives that they will complicate or hinder the attainment of common goals. The third dimension is task analysis/positive (TA+), defined as workers’ positive evaluation of the contextual, human, material, motivational, and structural resources that foster the collective’s operation. Lastly, the fourth dimension is task analysis/negative (TA−), defined as workers’ negative assessment of said contextual resources, which they believe will obstruct collective operations.

Based on this definition of collective efficacy, we advance our first hypothesis (H1): an intervention aimed at developing group competences among workers, supported by blended learning strategies, will have a positive impact on subjects’ assessment of the team’s group competences while also improving their evaluation of elements with an influence in their tasks, compared to a control group. We did not consider an intervention that directly targeted the analysis of elements with an influence on tasks, since the availability of these contextual resources depends on organizational conditions and regulations beyond our intervention capabilities. Nevertheless, in line with our theoretical framework, we still expect these perceptions to be influenced by the development of collective competences, considering that a better assessment and understanding of group competences will improve the usage and evaluation of these contextual resources, since both perceptions are related in each individual at an analytic-interpretative level (Goddard et al., 2000).

Secondly, in line with the conceptualization of competence, self-efficacy can be defined as the confidence in one’s ability to plan and carry out the actions necessary to handle different situations and goals (Bandura, 1995). For the work and organizational contexts, self-efficacy consists in workers’ beliefs regarding their capacities to attain work-related goals, including their skills for managing personal and contextual resources. In this sense, we implemented a theoretical model that defines self-efficacy in the workplace through five dimensions (Campos et al., 2021). The first is self-management, defined as an individual’s assessment of their ability to act on their own initiative and act strategically by prioritizing organizational tasks, schedules, and resources in order to attain specific goals. The second is transference, that is, an individual’s assessment of their ability to apply multiple sets of knowledge in various contexts and put them to use in the workplace with a view to achieving a given objective. The third dimension, metacognition, is a subject’s evaluation of them reflective capacity that enables them to decipher and recognize relevant information, anticipate certain events, construct pertinent hypotheses, manage tasks, and consider their strengths and weaknesses when performing a given task. The fourth dimension, meta-learning, is a subject’s evaluation of their ability to identify and develop strategies to achieve effective learning. Lastly, self-care is a subject’s assessment of their ability to identify and regulate factors that affect personal wellbeing, allowing them to implement strategies and personal resources to increase their wellbeing and work performance.

Based on this definition of self-efficacy, we proposed our second hypothesis (H2): the development of collective competences through blended-learning will have a positive impact on self-efficacy and its dimensions, compared to a control group. This hypothesis is supported by studies that evidence the relationship between collective and individual factors, particularly in the context of worker collaboration and professional development activities, where self-efficacy is influenced by social persuasion, vicarious experiences, and mastery experiences, which often involve group interactions, feedback, and collaborative efforts (Täschner et al., 2024). These findings suggest that group dynamics and collective activities play a significant role in shaping and enhancing individual self-efficacy among teachers.

As stated before, an intervention aimed at introducing new organizational practices in a work context, also has the expectation of contributing to the personnel wellbeing. In this area, it’s argued that there are two major traditions in the study of wellbeing: the hedonic tradition, centered on overall satisfaction, and the eudaimonic tradition, focused on personal development and goal attainment (Keyes et al., 2002). In this study, we adopt a eudaimonic view of wellbeing, considering its alignment with the development of collective competences in the individuals, and the consequent personal and organizational goals that these competences imply. Considering this definition of eudaimonic wellbeing, social wellbeing can be defined as individuals’ appraisal of them circumstances and role in society. The literature shows that it is a multidimensional construct, which continues to exist over time, and that people who enjoy more social wellbeing have feelings of belonging and solid social ties, trust both themselves and others, feel useful within the collective, have confidence in the future of society, are aware of their own potential, and regard the world as something full of meaning and objectives (Keyes, 1998). In this regard, socio-occupational wellbeing can be defined as people’s positive assessment of the organization for which they work, considering both cognitive and emotional aspects (Dagenais-Desmarais and Savoie, 2012; Bakker and Demerouti, 2018).

Socio-occupational wellbeing is defined through three factors: Social Belonging, which consists in workers’ positive assessment of the degree to which they feel attached to the organization, encouraging a feeling of usefulness and fidelity to it; Social Interaction, defined as workers’ positive assessment of the qualities of their coworkers and the organization as a social system, considering the quality of the bonds and interpersonal relationships established; and Social Comprehension, which consists in workers’ positive assessment of their understanding of the social and administrative functioning of the organization, which enables them to understand social ties and events in the workplace (Campos-Carreño et al., 2020). With respect to this concept, it has been noted that social support and work satisfaction influence socio-occupational wellbeing, promoting workers’ mental health an sense of belonging (Marrero and Carballeira, 2010). The social systems that encourage collaboration and joint problem resolution can help to increase both socio-occupational wellbeing and productivity (Cruz-Ortiz et al., 2013). Satisfactory workplace relationships and the ability to find a balance between one’s work and personal life are key determinants of socio-occupational wellbeing, whereas factors such as working long hours can have a negative impact on this aspect, especially in sectors such as mining (López-Mena and Campos-Álvarez, 2002). In sense of this definition of socio-occupational wellbeing, and these studies around what relates to its presence in workers’ perception, we proposed our third hypothesis (H3), stating that a group intervened in their collective competences, will show an improvement in the three dimensions of socio-occupational wellbeing, in comparison to a control group. Also, we advance our fourth hypothesis: (H4): the development of collective competences has a positive impact in the self-efficacy, collective efficacy and the social wellbeing on workers in the occupational context. Given this information, we defined our research objective: to evaluate how the development of collective competences, based on a blended learning methodology, could influence the variables self-efficacy, collective efficacy, and socio-occupational wellbeing, using a quasi-experimental sample of workers employed by a mining company in the Coquimbo Region. We established the following hypotheses based on what said information suggests regarding the outcomes of our analysis: Hypothesis 1: There are significant differences between the pre-test and post-test phase evaluation of collective efficacy for the intervention group, in contrast to the control group. Hypothesis 2: There are significant differences between the pre-test and post-test phase evaluation of self-efficacy for the intervention group, in contrast to the control group. Hypothesis 3: There are significant differences between the pre-test and post-test phase evaluation of socio-occupational wellbeing for the intervention group, in contrast to the control group. Hypothesis 4: The development of collective competences, on a blended learning methodology has a positive effect on each dimension of the constructs under study: self-efficacy, collective efficacy, and socio-occupational wellbeing in the quasi-experimental group.

## 2 Materials and methods

### 2.1 Design

We employed a quasi-experimental pre-test/post-test design. The study variables –Self-Efficacy, Collective Efficacy, and Socio-Occupational Wellbeing– were measured in a quasi-experimental group of subjects who participated in a training program aimed at building collective competences and in a control group of subjects who took part in the evaluations, but not in the training program.

We use the term “quasi-experimental” to indicate that the groups retained their natural composition intact instead of being rearranged for the study (Hernández-Sampieri and Mendoza, 2018), given the need to measure the variables according to the organizational structure that subjects naturally maintain in their workplace. For both groups, the variables were measured before the intervention and 3 months after the end of the intervention.

### 2.2 Participants

The sample comprised a total of 110 workers employed by a mining company that extracts copper and gold in the Coquimbo Region. The subjects belong to two groups: an intervention or quasi-experimental group (*n* = 73) and a control group (*n* = 37), both composed of Plant Operators and Supervisors belonging to the Grinding, Crushing, and Flotation units of the Operations division. Their ages range from 24 to 57 years, with an average of 36.7 (SD = 9). Table 1 specifies other demographic data and their distribution.

**Table 1:** Socio-demographic characteristics of the sample and their distribution in the quasi-experimental and control groups.

Variable		Intervention group		Control group		Total	
		N	%	N	%	N	%
Age	20–29 years	13	17.81	10	27.03	23	20.9
	30–39 years	33	45.21	17	45.95	50	45.5
	40–49 years	19	26.03	8	21.62	27	24.5
	50–59 years	8	10.96	2	5.41	10	9.1
Sex	Man	72	98.63	37	100	109	99.1
	Woman	1	1.37	0	0	1	0.9
Position	Operators	56	76.71	28	75.68	84	76.4
	Supervisors	17	23.29	9	24.32	26	23.6
Educational level	Secondary education (complete)	21	28.77	16	43.24	37	33.6
	Secondary education (incomplete)	20	27.4	6	16.22	26	23.6
	Technical Education Center	17	23.29	9	24.32	26	23.6
	Professional Institute	4	5.48	3	8.11	7	6.4
	University	11	15.07	3	8.11	14	12.7

### 2.3 Intervention design

The intervention implemented in this study consisted in a blended learning program aimed at developing collective competences which combined in-person and collaborative strategies with digital tools in complementary, personalized and collaborative spaces, which allowed the participants to freely interact with the professional team and with each other. The training process implied that the participants first completed the virtual courses, and then they assisted each correspondent in-person workshop. To prepare these workshops, in the stage prior to their implementation, a team –composed of two psychologists, one computer expert, and several company informants– was set up to develop, complement, and adjust the contents included in the intervention.

The intervention was conducted over a four-month period, with five in-person workshops being conducted which lasted approximately 6 h each. In parallel, during this period, we set up a virtual platform that was available around the clock, every day of the week, from the start of the intervention until its completion. The online platform had three virtual tutors available for users to contact from Monday to Friday during office hours.

This virtual platform, entitled “System for Developing and Evaluating Work-Related and Employability Competences” (Sistema de Desarrollo y Evaluación de Competencias Laborales y de Empleabilidad, SIDECOMP), was originally produced by the Human Capital Development Center of the Universidad de La Serena as part of CORFO – INNOVA project code 07CN13PXD-159. Among other contents, the platform has a section devoted to the development of collective competences. It was created using Moodle, a Learning Content Management System (LCMS) distributed under the GNU General Public License which offers great versatility when integrating various applications. We also utilized Moodle’s Learning Activity Management System (LAMS) functionalities to design, manage, and conduct online learning activities collaboratively within a visual environment. These contents were produced with a bottom-up approach, benefiting from the input of workers from various fields and experts in the generation of contents and learning objects. This methodology allows workers to be the protagonists of their own development, ensuring accessibility and continuity over time.

The platform is characterized by its intuitive visual interface, the quality of its content, the inclusion of a variety of activities, and the availability of content questionnaires, configured to offer immediate feedback. By facilitating synchronous and asynchronous interactions among participants, SIDECOMP can be regarded as a social venue for virtual learning, aimed at fostering the creation of communities, encouraging the use of collaborative work methodologies, and increasing co-responsibility. Two interactive functionalities were set up (“In Touch” [“Comunicados”] and “Connected” [“Conectados”]). The “Connected” functionality simplified participant-tutor and participant-participant communication and learning interaction through Zoom video calls and chats. The monitoring mode, employed by the virtual tutors, was essential, because it enabled them to track the participants’ individual and collective progress and boost their commitment to the process. The “In Touch” functionality allowed the participants to freely interact with each other, without direct supervision from the intervening team, enabling an informal and more friendly relationships, which ended in the organization of different out-of-work activities, such as football matches, online gaming, partying with each other, among other similar activities.

The content units available on the virtual platform focused on collaborative problem-solving and offered a number of motivational elements to encourage the participants to continue using the platform. These contents were built using knowledge validated in the field, provided by mining company informants. They were designed in line with strategies that matched those implemented in in-person activities, in order to ensure the integration and continuity of both learning modes, and without losing sight of the goal of improving the company’s productive processes. In-person workshops were defined as opportunities to gain a deeper understanding of a specific topic through personal exchanges among the participants. Interactivity is the fundamental characteristic of this teaching approach, which fosters the exchange of experiences, experimentation, and reflection among the participants. The main features of the workshops and the methodology implemented are the following: (1) they provide tools and strategies to improve the functioning of the system, expanding participants’ knowledge and enabling them to rehearse behaviors before transferring them to other contexts; (2) they stand out due to their active-participatory, experiential, and interactive approach, encouraging personal and collective goal-driven efforts and the search for quality; and (3) each virtual module has a workshop implemented by a development agent, whose objective is to build collective competences and generate an environment conducive to collaboration.

The contents of the in-person workshops focused on the following elements: (1) communication within work teams, which involves building up competences such as Expressing opinions assertively, Understanding others empathetically, and distinguishing and utilizing speech acts with a view to improving work performance and collaborative learning; (2) participation in work teams, which includes developing competences such as Participating actively in coordination processes, Establishing team objectives, Strategic planning and alignment, Solving and addressing conflicts, and Establishing control and follow-up mechanisms; and (3) negotiation, which involves building up the competences Identifying and mobilizing the fundamental aspects of a negotiation, Mastering win-win cooperative negotiation techniques, and Developing skills for negotiating at the social system level in order to foster collective development. Each workshop had a manual for the development agent in charge, offering flexible strategies relevant to the needs of the social system targeted by the intervention. Apart from these resources, work booklets and audiovisual resources were used to enrich the participants’ learning experience. Owing to these components, the workshops emerge as dynamic venues that promote interaction and competence development practically and collaboratively.

### 2.4 Ethical safeguards

For the present study, we signed collaboration agreements with the authorities of the company were the intervention was to be implemented. The workers who volunteered to participate in the study signed individual informed consent documents. This documentation explained aspects such as the objective of the study, how the participants’ rights would be safeguarded, how their identity and privacy would be protected, and how their data would be securely stored, certifying that they would only be used for research purposes.

### 2.5 Instruments

#### 2.5.1 Self efficacy scale

Instrument validated by Campos et al. (2021) for workplace use. It comprises 41 Likert items, with scores ranging from 1 (strongly disagree) to 4 (strongly agree). It is organized around five dimensions: self-management, transference, metacognition, meta-learning, and self-care.

The instrument meets reliability standards (*α* = 0.948).

#### 2.5.2 Collective efficacy scale

Instrument adapted and validated by Campos et al. (2020) for workplace use, based on the scale developed by Goddard et al. (2000). It is composed of 19 Likert items, with scores ranging from (strongly disagree) to 4 (strongly agree). It comprises four dimensions: group competence/positive (GC+), group competence/negative (GC−), task analysis/positive (TA+), and task analysis/negative (TA−). The instrument meets reliability standards (*α* = 0.96).

#### 2.5.3 Socio-occupational wellbeing scale

Instrument validated by Campos-Carreño et al. (2020) for workplace use; originally developed by Keyes (1998) and adapted and translated into Spanish by Blanco and Díaz (2005). It is composed of 13 Likert items, with scores ranging from 4 (strongly disagree) to 1 (strongly agree). It comprises three dimensions: social belonging, social interaction, and social comprehension. Like the previous instruments, this scale also meets reliability standards (*α* = 0.938).

### 2.6 Data analysis

To analyze and process the data, we used SPSS 29. We employed descriptive tests of central tendency (frequency, mean, and standard deviation) and inference, specifically Student’s t-test for unpaired and paired samples (Hernández-Sampieri and Mendoza, 2018). First, for the unpaired samples, we tested whether significant differences existed between the pre-test means of the intervention and the control groups. Then, for the paired samples, we analyzed whether significant differences existed between the pre-test and post-test measures of the quasi-experimental and the control groups. Lastly, we checked whether the unpaired samples exhibited significant differences between the post-test measures of both groups.

To evaluate the impact of the intervention on each dimension of the constructs under study: Self Efficacy, Collective Efficacy, and Socio-Labor Well-being, was performed paired t-tests and calculating the effect size (Cohen’s d), a pre-post analysis of the quasi-experimental group, following the technical and practical recommendations of the recent guide on effect sizes and their confidence intervals (Jane, 2024). A significance level of *p* ≤ 0.05 was adopted, a criterion commonly accepted in the social sciences to determine whether the observed changes are statistically significant (White et al., 2022).

## 3 Results

First, with respect to the participants, 97 out of 110 subjects who took the pre-test evaluation (88.2%) completed the post-test one. The deficit in the post-test sample appeared in the intervention group, here 60 of the 73 subjects (82.1%) responded to the post-test evaluation. The rate of participation remained the same for the control group.

The results presented in this section describe the average scores, the standard deviation (SD), and the differences between the pre-test and post-test averages of the intervention and control groups (Table 2). Student’s *t*-test for unpaired samples revealed the presence of significant differences between the pre-test and the post-test scores of the intervention group for all the study variables; however, it showed no significant differences in the control group. These significant differences found in the intervention group support our first three hypotheses (H1, H2, and H3), since they can be regarded as a consequence of a blended-learning program aimed at building up collective competences.

**Table 2:** Pre-test and post-test mean differences between the intervention group and control group, analyzed using paired samples *t*-test.

Variable	Group	Pre-test mean (SD)	Post-test mean (SD)	Mean difference
Self efficacy	Intervention group	145.31 (13.97)	150.67 (10.01)	4.87**
	Control group	143.05 (14.14)	145.22 (16.24)	2.17
Collective efficacy	Intervention group	61.57 (6.35)	70.78 (5.27)	8.81**
	Control group	60.65 (6.19)	61.46 (6.75)	0.81
Socio-occupational wellbeing	Intervention group	55.84 (9.41)	60.32 (5.66)	4.28**
	Control group	54.30 (10.28)	56.45 (8.77)	2.15

**p < 0.01. Pre-test sample: Quasi-experimental group (*n* = 73), Control group (*n* = 37). Post-test sample: Quasi-experimental group (*n* = 60), Control group (*n* = 37). SD, standard deviation.

Apart from the differences between the intervention group’s pre-test and post-test scores for each study variable, Student’s t-test for paired samples also revealed significant differences in the constitutive dimensions of each variable, as shown in Table 3.

**Table 3:** Results of pre-test and post-test student’s *t*-test in the intervention group.

Dimension	Pre-test		Post-test		Difference
	M	SD	M	SD	
Self efficacy	145.8	13.34	150.67	10.01	4.87**
Self-management	38.08	10.01	39.63	2.99	1.55**
Transference	32.35	3.17	33.48	2.51	1.13**
Metacognition	31.52	3.31	32.43	2.75	0.92**
Meta-learning	18.05	1.88	18.82	1.4	0.77**
Self-care	25.8	2.49	26.3	2.01	0.50**
Collective efficacy	61.97	6.15	70.78	5.27	8.81**
Group competence/positive	22.35	3.41	25.22	2.1	2.86**
Group competence/negative	16.5	2.86	19.93	1.87	3.43**
Task analysis/positive	12.73	1.71	13.57	1.44	0.83**
Task analysis/negative	10.38	2	12.07	1.74	1.68**
Socio-occupational wellbeing	56.03	9.73	60.32	5.65	4.28**
Socio-occupational Interaction	25.78	4.88	28.1	3.01	2.32**
Socio-occupational Belonging	17.15	3.15	18.37	1.76	1.22**
Socio-occupational comprehension	13.1	2.5	13.85	1.78	0.75**

*N* = 60; df = 59; **p < 0.01.

Similarly, in the control group, the results of Student’s *t*-test for paired samples revealed no statistically significant differences for any of the study variables or their associated dimensions (Table 4).

**Table 4:** Results of pre-test and post-test student’s *t*-test in the control group.

Variable	Pre-test		Post-test		Difference
	M	SD	M	SD	
Self efficacy	143.05	14.14	145.22	16.24	−2.17
Self-management	37.22	4.14	37.95	4.6	−0.73
Transference	31.65	3.49	31.86	4.52	−0.21
Metacognition	31.38	3.23	31.38	4.19	0
Meta-learning	17.59	2.33	18.22	2.04	−0.63
Self-care	25.22	3.04	25.81	2.58	−0.59
Collective efficacy	60.65	6.19	61.46	6.75	−0.81
Group competence/positive	21.95	2.98	22.3	3.74	−0.35
Group competence/negative	15.95	3.73	16.14	2.61	−0.19
Task analysis/positive	12.65	1.4	13	1.76	−0.35
Task analysis/negative	10.68	1.97	11.08	1.98	−0.4
Socio-occupational wellbeing	54.3	10.28	56.45	8.77	−2.15
Socio-occupational interaction	25	5.3	25.92	4.46	−0.92
Socio-occupational belonging	16.89	3.23	17.19	2.96	−0.3
Socio-occupational comprehension	12.73	2.81	13.35	2.15	−0.62

*N* = 60; df = 59.

The results of the effect size (Cohen’s *d*), in the quasi-experimental regarding of the five dimensions of the Self-Efficacy Scale, realize values ranging from moderate to large, according to recent empirical guidelines in social psychology (*d* ≈ 0.36 = medium, *d* ≈ 0.65 = large) (Lovakov and Agadullina, 2021).

Self-management achieved the largest effect size (*d* = 0.65), suggesting that it was the dimension most impacted by the intervention, reflecting significant progress in planning, self-regulation, and work performance organization skills. This was followed by Meta-learning (*d* = 0.55) and Transfer (*d* = 0.54), both dimensions associated with autonomous learning and the flexible application of knowledge (Table 5). Finally, Self-Care and Metacognition (both with *d* = 0.35) also showed significant improvements, although with moderate effects. These results confirm that the intervention was effective in strengthening individual competencies associated with Personal Effectiveness.

**Table 5:** Pre-post analysis by dimension of the self-efficacy scale (quasi-experimental group).

Dimension	Pre-test	Post-test	t.t	*p*-value	Cohen´s *d*
Self-management	37.37	39.56	5.59	<0.001	0.65
Meta-learning	17.88	18.85	4.73	<0.001	0.55
Transference	31.71	33.40	4.63	<0.001	0.54
Self-care	25.51	26.25	3.03	0.034	0.35
Meta-cognition	31.44	32.30	3.00	0.0037	0.35

With regard to the Collective Efficacy Scale, the results show a significant impact of the intervention in all its dimensions, with moderate and very large effect sizes (Table 6). The greatest variations were observed in the dimension of negative group competencies (CG−), which showed a large effect size (*d* = 1.39), suggesting a significant decrease in dysfunctional perceptions in collective work. This improvement could be associated with greater awareness of the factors that hinder collaboration within the team.

**Table 6:** Pre-post analysis by dimension of the collective efficacy scale (quasi-experimental group).

Dimension	Pre-test	Post-test	t.t	*p*-value	Cohen´s *d*
Group competence/negative	15.75	19.95	11.89	<0.001	1.39
Group competence/positive	22.01	24.86	8.91	<0.001	1.04
Task analysis/negative	10.82	12.01	5.32	<0.001	0.62
Task analysis/positive	12.70	13.47	4.44	<0.001	0.52

Secondly, the dimension of positive group competencies (CG+) also showed a significant change (*d* = 1.04), which would indicate a strengthening of collaborative practices and attitudes among workers. For their part, the dimensions of analysis of negative elements (AT−) and analysis of positive elements (AT+) showed moderate effect sizes (*d* = 0.62 and *d* = 0.52, respectively), suggesting that the intervention mainly favored the perception of the group’s own capabilities.

Regarding the perception of external factors that affect collective work, a moderate effect is observed, which responds to the characteristics of the intervention process implemented, which focuses both on the group’s own capabilities and on the articulation and coordination with external elements that influence the tasks.

The impact of the intervention on the Socio-occupational Well-being Scale (Table 7), measured using the effect size (Cohen’s d), reveals that the three dimensions evaluated show improvements after the implementation of the intervention. The Socio-Occupational Interaction dimension shows the greatest change (*d* = 0.63), suggesting a significant strengthening of the quality of interpersonal relationships in the workplace, a key element for cohesion and collaboration in work teams. This is followed by Socio-occupational Belonging (*d* = 0.54) and Socio-occupational Comprehension (*d* = 0.46), both with moderate effects. These results reflect the positive effect of the intervention on workers’ socio-labor perceptions, especially in those dimensions that promote bonds and a sense of belonging.

**Table 7:** Pre-post analysis by dimension of the socio-occupational wellbeing scale (quasi-experimental group).

Dimension	Pre-test	Post-test	t.t	*p*-value	Cohen´s *d*
Social interaction	25.23	28.04	5.37	<0.001	0.63
Social belonging	16.90	18.30	4.63	<0.001	0.54
Social comprehension	12.81	13.84	3.96	<0.001	0.46

**Figure 1:**
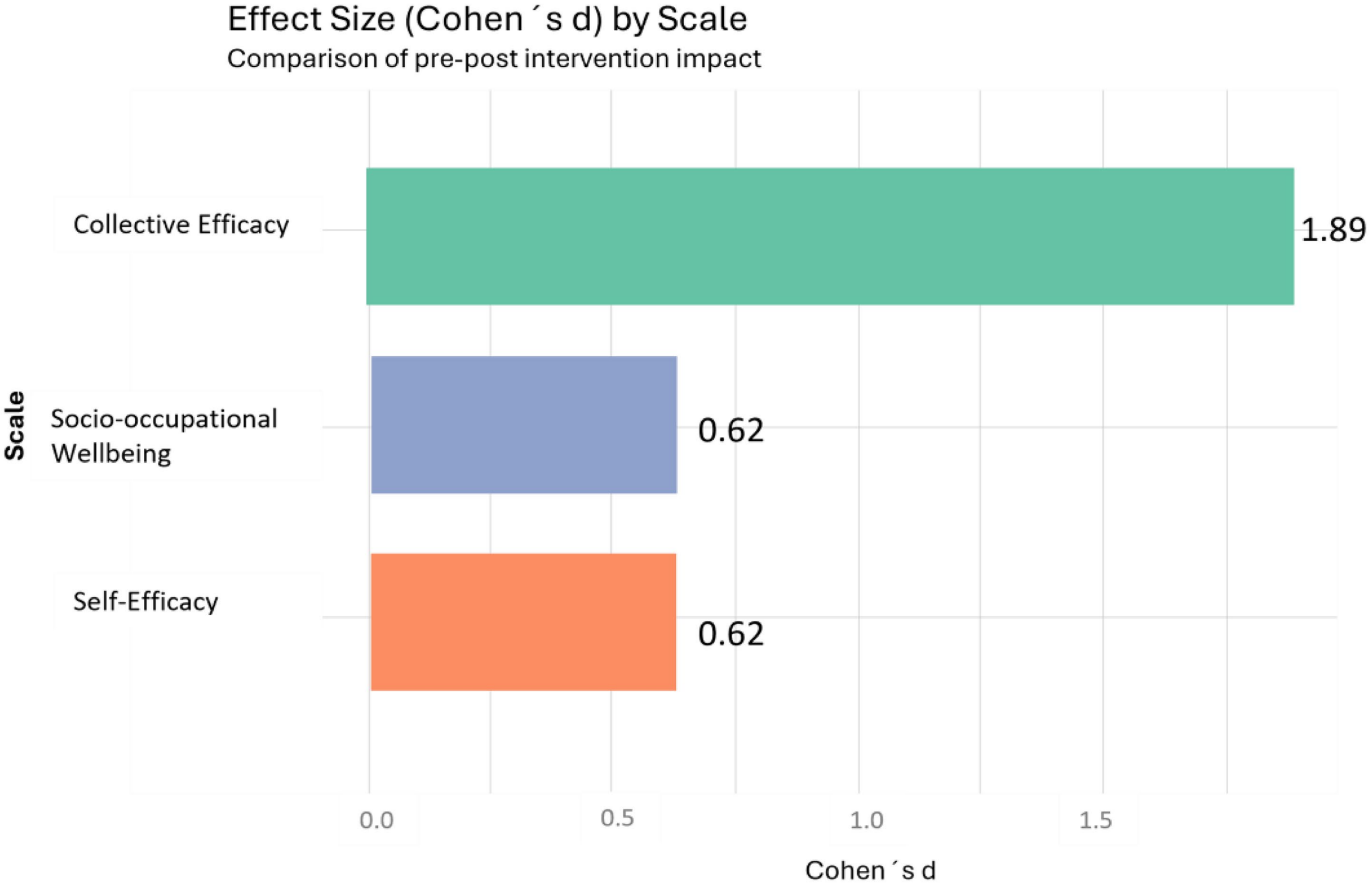
Comparison of pre and post intervention impact by Scale (quasi-experimental group).

In summary, the three constructs under study and their associated dimensions show significant improvements post-intervention in the quasi-experimental group with a high level of statistical significance *p* < 0.001. Social interaction showed the greatest effect, reflecting the strengthening of work relationships and a greater willingness to collaborate. These findings allow us to conclude that the intervention has an impact at both the individual and group levels, mobilizing collective competencies that are key to effective performance in work contexts. The results shown confirm the fourth and final hypothesis (H4) of the study. The intervention has a positive effect in each of the variables in study Self-Efficacy, Collective Efficacy and Socio-occupational Wellbeing (Table 8 and Figure 1) and its dimensions.

**Table 8:** Effect size (Cohen’s *d*) and statistical results by scale (quasi-experimental group).

Scale	Pre-test mean	Post-test mean	t.t	*p*-value	Cohen´s *d*
Self-Efficacy	143.90	150.36	5.26	<0.001	0.62
Collective Efficacy	60.64	70.29	16.17	<0.001	1.89
Socio-occupational Wellbeing	54.95	60.18	5.26	<0.001	0.62

## 4 Discussion and conclusion

The results show that the collective skills development intervention had a significant impact on various dimensions of self-efficacy, particularly self-management, with an effect size greater than *d* = 0.65. This finding reflects a strengthening of individuals’ ability to regulate their behavior, set goals, manage their emotions, and maintain their motivation in the face of work challenges. From the perspective of Bandura’s (2000) Social Cognitive Theory, self-management represents a central component of human agency, and its strengthening acts as a catalyst for self-regulation and autonomous performance. Furthermore, in the organizational context, self-management is related to the ability of workers to contribute actively and healthily to teamwork, reducing friction and promoting collaborative dynamics (Salanova and Llorens, 2016).

The moderate improvements observed in the dimensions of transfer and meta-learning (*d* ≈ 0.55) suggest that participants not only developed skills to apply learning in different contexts, but also reflective competencies that enable them to optimize their own learning processes. These skills are fundamental for continuous learning and adaptability in complex work environments, as demonstrated by recent meta-analyses on self-regulated learning interventions (Van Alten et al., 2022), which show sustained improvements in academic and professional performance. Likewise, both dimensions are closely linked to the development of personal resources, acting as protective mechanisms against high psychosocial demands.

The dimensions of self-care and metacognition had more modest but still relevant effects (*d* ≈ 0.35). This indicates that the intervention also succeeded in promoting individual well-being and cognitive regulation practices, although more specific or sustained strategies may be required over time to generate more robust effects. Taken together, these results confirm that interventions focused on developing collective competencies can have a significant impact on perceptions of self-efficacy, strengthening cross-cutting skills that are essential for both individual well-being and collective performance in organizational contexts.

In terms of social and occupational well-being, the results show that the collective skills development intervention had a positive impact, especially in the relational dimensions (interaction and social and occupational belonging). This is consistent with the approach to social well-being at work proposed by authors such as Keyes (1998) and Salanova and Llorens (2016), who emphasize the importance of the quality of interpersonal relationships and the sense of belonging for psychological health and sustainable productivity. Likewise, the findings support the relevance of implementing strategies that strengthen social capital in organizations, understanding that well-being is not only individual, but also collective and relational.

The findings derived from the effect size analysis indicate that the collective skills development intervention had a moderate to high impact on the dimensions that make up socio-occupational well-being. In particular, the socio-occupational interaction dimension showed the most pronounced effect (*d* ≈ 0.62), suggesting that the intervention favored the quality of labor relations, improving cooperation, effective communication, and mutual respect within teams. This result is in line with previous studies that highlight how interventions aimed at improving collective competencies, such as shared leadership, empathy, or coordination, have positive effects on interpersonal dynamics and group cohesion (Hakanen et al., 2021; Salanova and Llorens, 2016). On the other hand, the socio-occupational belonging dimension also showed a relevant effect size (*d* ≈ 0.54), indicating an improvement in the sense of inclusion, recognition, and subjective value of individuals within the work group. From the social well-being approach proposed by Keyes (1998), this dimension is central to the development of healthy environments, as it strengthens the emotional bond with the organization and promotes a positive collective identity. At the empirical level, recent studies indicate that when workers feel that they are an active and valued part of their team, levels of psychological well-being, engagement, and organizational commitment increase (Huertas-Valdivia et al., 2023).

The socio-occupational understanding dimension showed a slightly smaller effect size (*d* ≈ 0.44), although still within the moderate range. This suggests progress in the ability to interpret social norms, roles, and shared expectations in the workplace, which can facilitate coexistence and adaptability within teams. These social skills are essential for preventing and resolving conflicts and promoting a positive psychosocial climate (Sonnentag, 2020). Overall, the results support the effectiveness of collective interventions not only in terms of performance and efficiency, but also in strengthening relational and social well-being at work, which is consistent with contemporary models of healthy and resilient organizations (Salanova et al., 2013; Bakker and Oerlemans, 2016).

The study analyzed differences between the variables self-efficacy, collective efficacy, and socio-occupational wellbeing in a quasi-experimental sample of workers, seeking to examine the effects of an intervention aimed at improving the collective competences of a group of employees from a mining company. Student’s *t*-tests made it possible to establish that, after the implementation of blended learning strategies, the intervention group exhibited significant improvements in the three study variables, including their respective dimensions; by contrast, the control group showed no significant differences. These results indicate that, as subjects develop collective competences and manage to apply them in the workplace, they attain more collective efficacy, develop greater self-efficacy, and perceive a higher degree of socio-occupational wellbeing. Thus, our findings highlight the relevance of developing work skills of an interpersonal and collective nature; furthermore, they draw attention to the need to furnish organizational environments with the necessary spaces and resources to promote collaborative work strategies. This is consistent with what similar studies have shown, as the literature stresses the dynamic impact of collective efficacy, its long-term effects, and the inclusion of virtual spaces as a way for team members to interact (McLarnon and Woodley, 2021).

More specifically, the results enable us to assert that the development of collective competences prompts subjects to have a more positive assessment of their colleagues’ competences and the contextual resources that their team can employ to fulfill its tasks, with both these evaluations belonging to the positive dimensions of collective efficacy (Goddard et al., 2000; Campos et al., 2020). Likewise, perceived conflict regarding these dimensions, that is, the view that one’s team lacks competence or that contextual resources are insufficient, decreases when collective competences are developed in a team. These results support the findings of similar studies which single out collaborative functioning in work settings as a factor that increases the likelihood of finding solutions and making use of opportunities, promotes a change toward a collective perspective that fosters a systemic understanding of one’s environment (Muzard, 2012), and entails a dynamic.

The literature highlights that environments that promote mutual support and collaborative engagement not only improve mental health and job satisfaction but also drive organizational commitment and performance (Eby et al., 2005). The findings also suggest practical implications for organizational development and training programs. By fostering a culture of collaboration and continuous professional development through blended learning strategies, organizations can simultaneously enhance collective competences, self-efficacies, and socio-occupational wellbeing, leading to a more engaged and productive workforce (Salas et al., 2008). Future research should continue to explore these relationships, particularly the mechanisms through which collective competences influence individual and organizational outcomes, to further validate and expand upon these findings.

Our results also highlight the importance of expansive transitions toward advanced collaborative practices, which emphasizes the need of a progressive movement through three modes of work: coordination, cooperation and reflective communication (Engeström, 1999). This was achieved with the relation of the blended-learning strategies, where the social interactions supported in both the virtual platforms and the in-person workshops, aimed to foster each one of these working modes; considering the progress of the workers as an expansive transition. In this sense, we can argue that the development of collective competencies, and the subsequent improvement of self-efficacy, collective efficacy, and socio-occupational well-being, are key elements to be considered in relation to Engeström’s theory (Campos et al., 2020). Furthermore, through our intervention background and implementations, we propose a concept which can be understanded as a fourth mode of expansive working, the collaborative action in context, representing the transference of knowledge and competences to the real, in-place working environment.

The integration of ICTs in organizations significantly enhances productivity and operational flexibilities (Hervas-Oliver et al., 2017), with blended learning playing a crucial role in this process. ICTs reduce administrative burdens, reconfigure work groups, and promote employee autonomy and skill development (Medzo-M’engone et al., 2019). Our results highlight the importance of leveraging technological advancements and remote work to improve efficiency, emphasizing that digital solutions can effectively address various organizational needs, in both individual and group levels. Blended learning combines in-person and online training methods, fostering a flexible and dynamic learning environment that enhances job satisfaction and performance, aligning with organizational goals of productivity and efficiency (Hrastinski, 2019; Vallée et al., 2020).

With respect to the practical implications of the study, it is worth noting that the improvement measured in the study variables stems from a concrete intervention methodology, based on a constructivist and psychosocial approach aimed at strengthening collective competences and facilitated by the implementation of blended-learning technologies. The interventions were conducted in a participatory and reflective manner, fostering collaboration among group members. The methodology emphasized the importance of the activity, subjects, learning objects, and real work contexts involved, adopting a bottom-up approach and promoting situated learning. Collaboration blurred the hierarchical distances between workers and supervisors, promoting horizontal interactions. In this context, we must highlight the relevance of sustainability in the intervention actions that help participants to shift their perspective and those that contribute to social responsibilities. This environment is articulated around the acceptance of heterogeneity in competence-related performances.

Regarding the above, traditional organizations with strong hierarchical structures can hinder the development of collective competences by prioritizing routinization and individual performance over collaboration and flexibility (Argyris, 2009). By contrast, more flexible, but strongly competitive organizations can limit collaboration due to an excessive emphasis on individual competition (Engeström, 2008). Collaborative functioning in the workplace requires promoting distributed collective competences, assessing the context, and overcoming potential organizational barriers collaboration and innovation. The implementation of processes that foster reflective communication and collaborative action in context can significantly contribute to human development and work productivities. With respect to the limitations of the study, we must point out that our methodological design was quasi-experimental, as the subjects who comprised the intervention and control groups were not randomly distributed in order to keep work structures and relational dynamics intact for later evaluation and analysis. Since this methodological approach limits the generalizability of the results, it is important for future studies to employ larger randomized samples.

## Data Availability

The raw data supporting the conclusions of this article will be made available by the authors, without undue reservation.
